# Amyloid Light-Chain (AL) Amyloidosis of the Trachea Associated With an Indolent B-cell Neoplasm

**DOI:** 10.7759/cureus.53074

**Published:** 2024-01-27

**Authors:** Anup Kumar Trikannad, Asis Shrestha, Sruthi Vellanki, Hira i Cheema, Tanvi H Patel, Ramya Bachu, Shobhit Sharma, Susanne K Jeffus, Sharmilan Thanendrarajan

**Affiliations:** 1 Internal Medicine, University of Arkansas for Medical Sciences, Little Rock, USA; 2 Radiology, University of Arkansas for Medical Sciences, Little Rock, USA; 3 Pathology, University of Arkansas for Medical Sciences, Little Rock, USA; 4 Hematology and Oncology, University of Arkansas for Medical Sciences, Little Rock, USA

**Keywords:** tracheal stenosis, small cd5-positive monotypic b-cell population, localized tracheal al amyloidosis, amyloidosis (al), b cell neoplasm

## Abstract

We report the case of a 66-year-old woman who was diagnosed with localized tracheal amyloid light-chain (AL) amyloidosis caused by an underlying B-cell neoplasm. The diagnosis was confirmed through subsequent bronchoscopy and biopsies; however, she experienced a challenging episode of hypoxic respiratory failure that required intervention. Repeat bronchoscopies showed persistent subglottic stenosis and tracheobronchomalacia, which led to tracheal debulking surgery and additional interventions. The patient's treatment began with rituximab, zanubrutinib, and dexamethasone with outpatient follow-up. The rarity of tracheobronchial amyloidosis and its connection to B-cell malignancies are highlighted, emphasizing the challenges in diagnosis and the importance of tailored treatment strategies. The patient's clinical course, characterized by atypical respiratory symptoms, delayed diagnosis, and an evolving treatment approach, underscores the complexities of managing such a rare and intricate case.

## Introduction

This case report details the diagnostic journey of a 66-year-old female with a medical history of hypertension, gastroesophageal reflux disease (GERD), obstructive sleep apnea (OSA), and a maternal history of lung cancer. She struggled for a decade with a cough of indeterminate cause and shortness of breath, which led to a pulmonology referral.

The diagnostic process involved a comprehensive array of imaging and bronchoscopic studies, revealing subglottic stenosis, tracheobronchomalacia, and nodular lesions within the trachea. A biopsy of the trachea confirmed the presence of kappa-type light-chain amyloid deposition, a condition characterized by the accumulation of monoclonal immunoglobulin light-chain fragments forming amyloid fibrils [1]. The rarity of tracheobronchial amyloidosis and its connection to B-cell malignancies serve to emphasize the challenges in diagnosis and the importance of tailored treatment strategies.

## Case presentation

The patient is a 66-year-old female who presented with persistent cough and shortness of breath for the past decade and was evaluated by multiple physicians without a definitive diagnosis. Her mother had a history of lung cancer and died at the age of 47 years. The patient denied any history of smoking, alcoholism, or weight loss.

**Figure 1:**
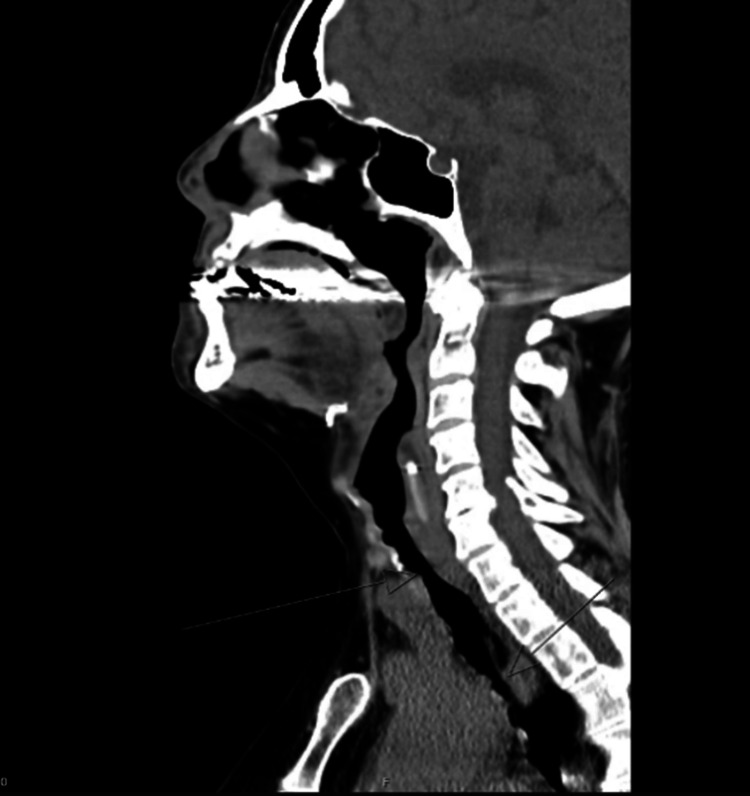
CT image of soft tissue neck without contrast (sagittal view) The image is showing multifocal areas of mucosal thickening in the subglottic region and in the trachea.

**Figure 2:**
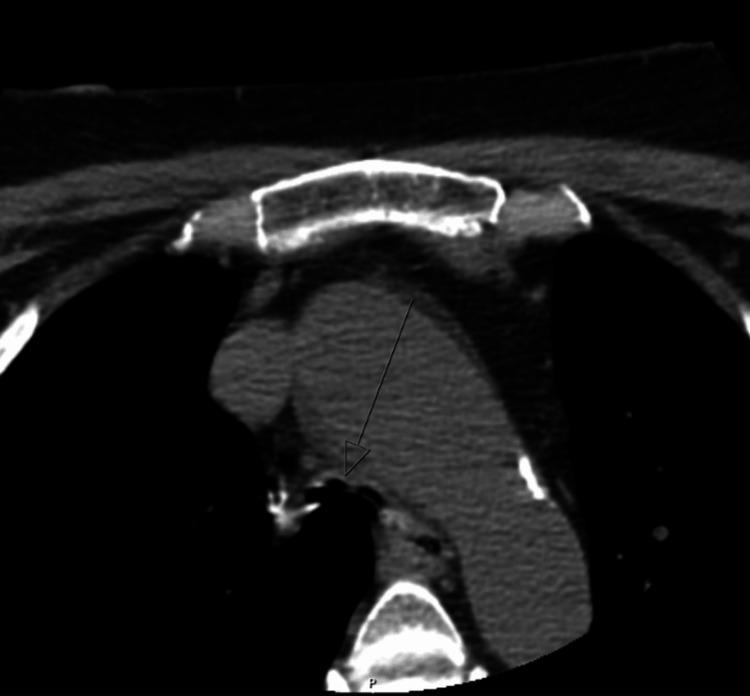
CT image of soft tissue neck without contrast (axial view) The image is showing stenosis of the trachea.

Before the case was transferred to our myeloma clinic, she was referred to a pulmonologist who conducted a neck computer tomography scan, which revealed subglottic stenosis with posterior tissue obstructing the subglottic area, along with thickening behind the subglottic and esophageal area (Figures 1, 2).

**Figure 3:**
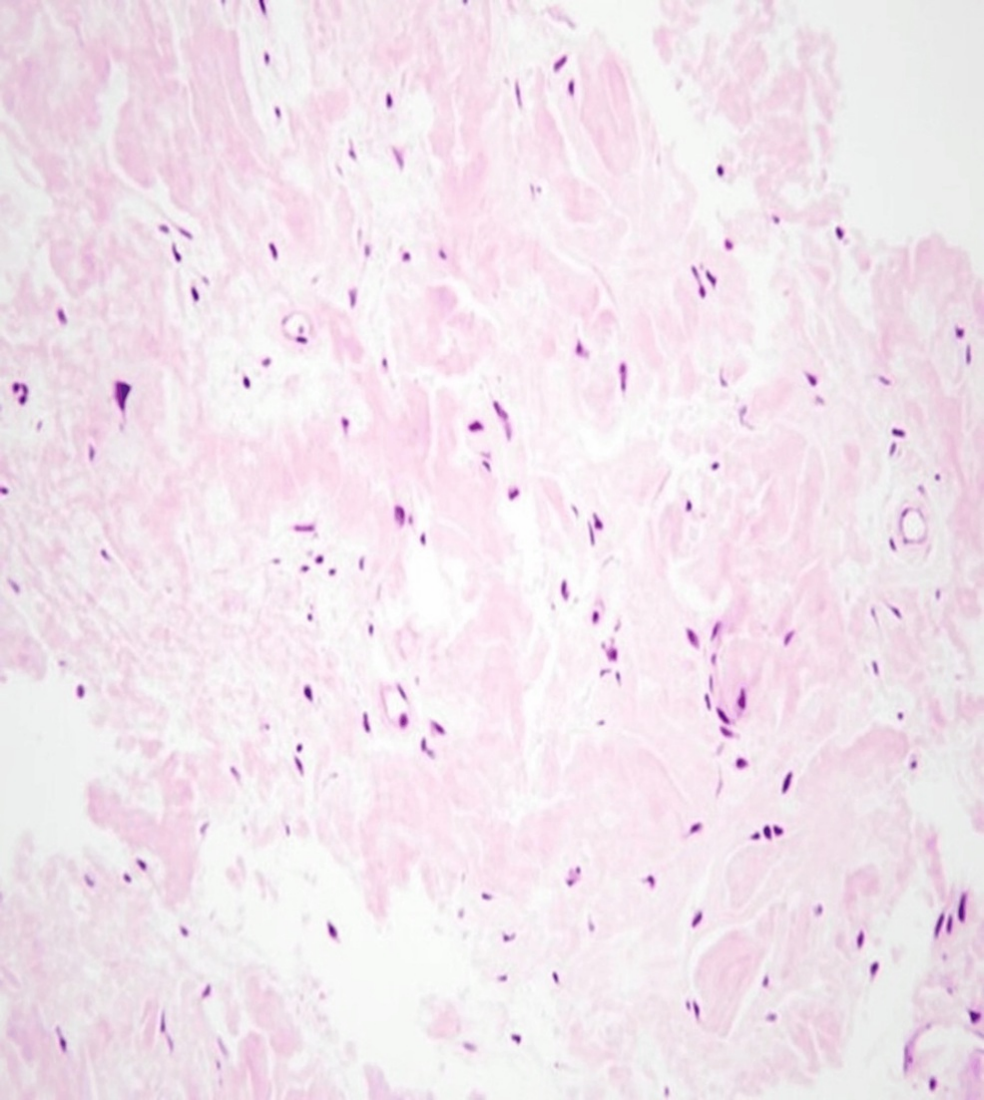
Histopathology image (H&E stain, 200X magnification) The biopsy is entirely composed of eosinophilic material with an amorphous, glassy appearance.

**Figure 4:**
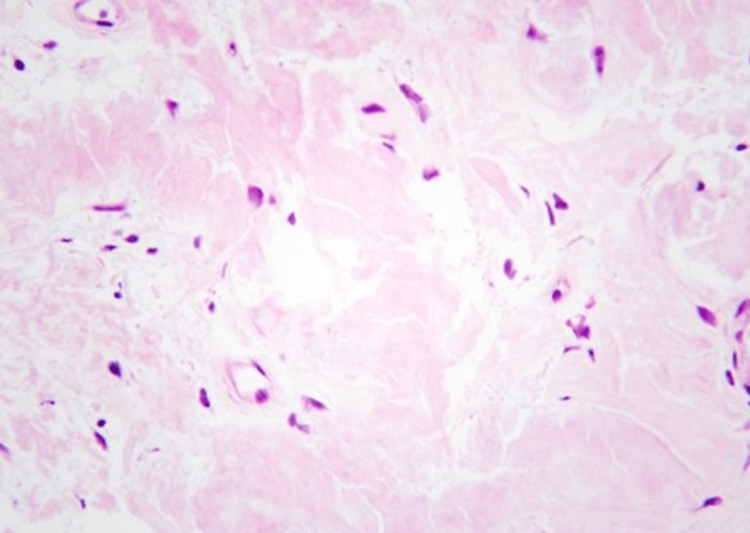
Histopathology image (H&E stain, 400X) Higher power magnification image.

**Figure 5:**
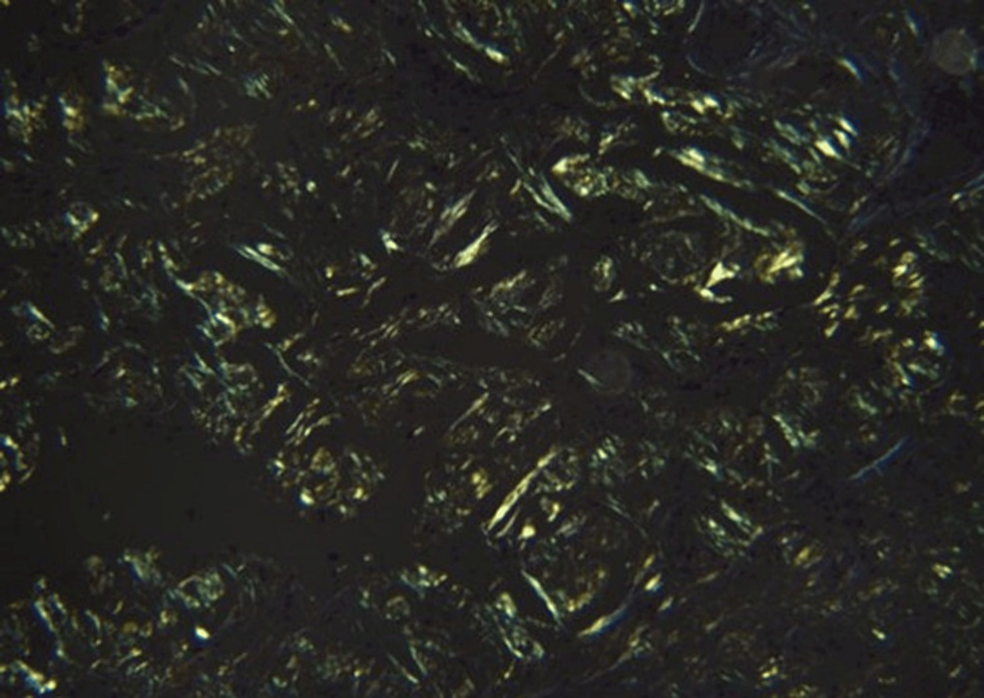
Histopathology image (Congo red stain, 100X magnification) The image shows apple green birefringence on polarization. This staining pattern supports the presence of amyloid.

Bronchoscopy identified multiple nodular lesions scattered throughout the upper two-thirds of the trachea. Biopsy results showed reactive respiratory mucosa with chronic inflammation and amyloid deposition revealed by Congo red staining (Figures 3-5).

A subsequent bronchoscopy showed 75% to 80% obstruction, and argon plasma coagulation with tracheal debulking was performed. Laboratory tests (Table 1), including white blood cell count, neutrophils, lymphocytes, creatinine, sodium, potassium, calcium, C-reactive protein, and liver enzymes (aspartate aminotransferase (AST) and alanine aminotransferase (ALT)), were within normal ranges. Serum and urine protein electrophoresis showed no monoclonal protein. Serum immunofixation revealed a faint immunoglobulin (Ig)G kappa band, with an increased kappa light chain and kappa/lambda ratio. Immunoglobulin levels were normal. Bone marrow examination showed no morphological signs of plasma cell neoplasm, but a small CD5-positive monotypic B-cell population (1%-2% of analyzed events) was detected by flow cytometry. This finding raised the possibility of chronic lymphocytic leukemia/small lymphocytic lymphoma (CLL/SLL) or monoclonal B-cell lymphocytosis. Minimal residual disease testing for atypical plasma cells was negative by flow cytometry. MRI studies for multiple myeloma revealed no focal lesions, except for degeneration in the lumbar spine with a hemangioma at the T10 level and cervical spine degeneration. A few scattered cervical, axillary, and pelvic lymph nodes were also seen. Positron emission tomography (PET) scan results showed no definitive focal lesions above the background marrow.

**Table 1:** Laboratory investigation results

Laboratory Investigation	Value	Reference Range
White Blood count	6.45 K/µL	3.60-9.50K/µL
Neutrophils	3.45 K/µL	1.40 K/µL -6.00 K/µL
Lymphocytes	2.41 K/µL	1.20 K/µL -3.40 K/µL
Creatinine	0.9mg/dl	0.4-1.0mg/dl
Sodium	140mmol/L	135-145 mmol/L
Potassium	3.6 mmol/L	3.5-5.1 mmol/L
Calcium	9.5mg/dL	8.6-10.2 mg/dL
C-reactive protein	<5.00 mg/L	0-10 mg/L
Aspartate Aminotransferase	32IU/L	15-41 IU/L
Alanine Transaminase	29IU/L	4-45 IU/L
Kappa Light Chain	7.48 mg/dL	0.33-1.94 mg/dL
Kappa/Lambda Ratio	5.02	0.26-1.65

The patient was diagnosed with localized tracheal amyloid light-chain (AL) amyloidosis due to an underlying B-cell lymphoma. Systemic treatment was considered with the aim of reducing light chains to control the disease and possibly achieve remission. She was started on intravenous rituximab (375 mg/m^2^) as a single agent, along with dexamethasone (20 mg) weekly. After two doses of treatment, the patient experienced an unresponsive episode with hypoxic respiratory failure requiring intubation complicated by tracheal stenosis. Repeat bronchoscopy showed subglottic stenosis and tracheobronchomalacia, and multiple tracheal nodules were resected. Stenting was not done, and a repeat rigid bronchoscopy has been planned. The patient was started on dexamethasone for tracheal stenosis, which was gradually weaned with a prednisone taper. She underwent two rounds of rituximab treatment. However, a setback occurred when her insurance denied additional rituximab treatment. Consequently, she commenced a regimen of zanubrutinib (160 mg) orally twice daily. Additionally, she underwent another argon plasma coagulation during her repeat bronchoscopy to further address subglottic stenosis.

## Discussion

AL amyloidosis results from tissue accumulation of monoclonal immunoglobulin light chain fragments that form amyloid fibrils. Although the majority of cases are linked to a plasma cell disorder in the bone marrow, both systemic and localized AL amyloidosis have been associated with B-cell lymphoproliferative disorders [1-4]. Even a small B-cell clone population is able to synthesize sufficient monoclonal protein to aggregate and form amyloid fibrils, causing organ damage or dysfunction [5].

Amyloidosis due to low-grade lymphoma has been broadly classified into three main groups: IgM-associated, Sjogren syndrome-associated, and localized lymphoma-related amyloidosis [3,4,6]. Non-IgM-related AL with lymphoma is commonly associated with marginal zone/MALT lymphoma [4,7,8] but has also been associated with CLL/SLL [8,9], aggressive lymphomas such as diffuse large B-cell lymphoma [3,8] and localized B-cell neoplasia [10]. Non-IgM-associated AL may present with localized amyloid deposits at the lacrimal gland, breast, lung, stomach, and lymph nodes [4,11].

In general, localized pulmonary amyloidosis can present in three forms: 1) nodular parenchymal amyloidosis, 2) diffuse parenchymal or alveolar septal amyloidosis or 3) tracheobronchial amyloidosis [12]. Tracheobronchial amyloidosis, as demonstrated in this case, is an uncommon form of localized amyloidosis and typically presents after the fifth decade of life [13,14]. B-cell neoplasms are more frequently associated with nodular rather than tracheobronchial amyloidosis [15,16].

Tracheal amyloidosis and non-IgM-associated lymphoma that causes amyloidosis are separate rare events. In an observational study by Basset et al., only one out of 36 patients with AL amyloidosis with non-lymphoplasmacytic lymphoproliferative disorders had tracheobronchial involvement [8]. In a study by Rech et al., two in 17 patients with tracheobronchial amyloidosis had hematological malignancies-one had multiple myeloma, and the other had chronic lymphocytic leukemia) [15]. 

Our case represents a rare diagnosis of lymphoma-associated tracheal AL amyloidosis. This condition has been linked with tracheobronchopathia osteoplastica, defined by calcified or cartilaginous submucosal nodules within the airways [17]. Our patient had nonspecific respiratory symptoms that remained indeterminate for about a decade. Symptom progression led to a referral to a pulmonologist and a subsequent CT scan of the patient’s neck that revealed subglottic stenosis. Patients with tracheobronchial amyloidosis are known to exhibit nonspecific symptoms related to a narrowing of the upper airway due to amyloid deposits. These symptoms may include cough, dyspnea, hoarseness, stridor, and dysphagia. Often, patients are initially assessed for common conditions such as asthma, chronic obstructive pulmonary disease (COPD), malignancy, and other lung diseases, resulting in delayed or misdiagnosis [12]. Though our patient was a non-smoker, the proportion of smokers is higher in tracheobronchial pattern compared to other subtypes of lower respiratory tract amyloidosis [15].

Tracheobronchial amyloidosis has been rarely reported with B-cell neoplasms. We identified a similar case of tracheobronchial amyloidosis reported by Borie et al. that was associated with local B-cell clonal proliferation [13]. The case was a 41-year-old female who presented with diffuse involvement of tracheobronchial mucosa, positive for lambda light chain antibody and a dominant B-cell clone confirmed by B-cell clonality analysis of the tracheobronchial sample. In our case, the tracheal amyloidosis was associated with a CD5-positive, undifferentiated, B-cell neoplasm. Non-Hodgkin lymphoma-associated AL can have an IgM paraprotein, but this was not observed in our patient [11].

There is no definitive consensus on treating lymphoma-associated AL amyloidosis, but the main treatment objective is to eliminate the monoclonal plasma cell or underlying B-cell clone responsible for the excessive production of the toxic amyloid light chain. Jaccard et al. reported that high-dose chemotherapy followed by autologous hematopoietic stem-cell transplantation provides higher response rates and better overall survival than standard chemotherapy in AL amyloidosis, but these two strategies have not been compared in a randomized study [18]. Achievement of hematological remission by reducing the pathologic light chain level produced by the lymphoma has been associated with better progression-free survival [3]. Most systemic therapeutic approaches for B-cell lymphoproliferative disorder-associated amyloidosis involve anti-CD20 monoclonal antibody treatment alone or in combination with chemotherapy, [3] but the management of localized tracheobronchial amyloidosis is usually largely dependent upon symptoms. Endobronchial management options may include debridement, laser ablation, balloon dilatation, and stent placement. With localized tracheal involvement of lymphoma-AL in our case, the patient initially underwent laser ablation with local excision but continued to develop tracheal stenosis, requiring repeated resection of the tracheal lesions. Local excisions for tracheobronchial amyloidosis are usually temporarily effective and have the chance of multiple recurrences [19].

Our patient was started on rituximab, which was later stopped due to insurance denial and is currently on zanubrutinib systemic monotherapy to suppress the B-cell clone that is producing amyloids, and she will be monitored in our clinic for response. In a similar case report by Borie et al., the patient was treated with rituximab and achieved stable disease; however, the B-cell clone was still detectable in tracheal biopsies after a year. This finding suggests rituximab alone may not be sufficient to clear the monoclonal B-cell population [13]. However, there is insufficient literature and evidence to fully understand the disease course, and therefore, treatment strategies have to be individualized for each patient. 

Treatment guidelines for tracheobronchial amyloidosis remain a challenge. This could mean combining endobronchial management of complications along with an etiological therapy targeting the B-cell clone, as well as antibodies to the serum amyloid component to increase the clearance of the insoluble protein fibrils [20]. We believe that literary reports such as ours trigger further intellectual thought processes and help to answer unsolved questions.

## Conclusions

Amyloidosis could be linked to a plasma cell disorder or B-cell lymphoproliferative disorders and could cause various symptoms depending on organ involvement. Herein, we report a rare case of a localized tracheal AL amyloidosis associated with B-cell neoplasm causing subglottic stenosis. Symptoms can vary from nonspecific respiratory symptoms to respiratory failure, and patients can be misdiagnosed for other conditions such as OSA or GERD. Bronchoscopy can help to visualize obstructive lesions, and biopsy can aid in diagnosis. A bone marrow exam can also help differentiate between B-cell lymphoproliferative disorder and plasma cell disorder to define the primary cause of amyloidosis. Management can include systemic approaches with anti-CD 20 monoclonal antibodies alone or in combination with chemotherapy, as well as localized measures such as debridement, laser ablation, balloon dilation, or stent placement. As there is no definitive consensus on the treatment of lymphoma-associated AL amyloidosis and because the condition can cause significant morbidity and mortality, clinicians should be vigilant in devising a therapy plan as well as specify regular follow-up to determine the response rate. More data, including long-term follow-up, is needed to understand the disease course, prognosis, and response to treatments.
